# hsa_circ_0001377 promotes hepatic fibrosis by sponging let-7a-5p and targeting TGFBR1, Smad2, and Col1a2

**DOI:** 10.3389/fphar.2026.1882205

**Published:** 2026-10-02

**Authors:** Jiaming Zhou, Jiaxuan Liu, Dikun Wu, Junjie Ding, Binbin Li, Hongyu Yu

**Affiliations:** 1 Department of Pathology, Medical School of Nantong University, Nantong, Jiangsu, China; 2 Department of Pathology, Second Affiliated Hospital of Naval Medical University, Shanghai, China

**Keywords:** hepatic fibrosis, hepatic stellate cells, hsa_circ_0001377, let-7a-5p, TGFBR1, Smad2, Col1a2

## Abstract

**Background**: Hepatic fibrosis (HF) is primarily driven by the activation of hepatic stellate cells (HSCs) and is characterized by excessive extracellular matrix (ECM) accumulation. Circular RNAs (circRNAs) have recently gained increasing attention in HF research.

**Methods**: RNA sequencing and bioinformatic analyses revealed elevated hsa_circ_0001377 expression in human HF tissues. Its circular structure and subcellular localization were identified using Sanger sequencing, RNase R treatment, and fluorescence *in situ* hybridization (FISH). Gain- and loss-of-function experiments were conducted to elucidate the role of hsa_circ_0001377 in the human HSC line LX-2. Furthermore, the *in vitro* and *in vivo* effects of the hsa_circ_0001377/let-7a-5p/TGFBR1, Smad2, and Col1a2 regulatory axis were investigated using cytological assays and a bile duct ligation (BDL)-induced mouse model of HF.

**Results**: hsa_circ_0001377 was significantly upregulated in the circRNA expression profile of human HF tissues. Its circular structure was validated through Sanger sequencing and resistance to RNase R digestion. FISH and co-staining with α-SMA demonstrated that hsa_circ_0001377 was abundantly expressed in activated HSCs (aHSCs; α-SMA-positive cells). Gain- and loss-of-function assays were performed using LX-2 cells with stable hsa_circ_0001377 overexpression and a specific siRNA targeting this circRNA. hsa_circ_0001377 overexpression promoted LX-2 cell activation, proliferation, migration, and G1/S-phase transition, whereas its knockdown produced the opposite effects. qRT-PCR, AGO-RIP, dual-luciferase reporter, and miRNA pulldown assays confirmed that hsa_circ_0001377 acts as a molecular sponge for let-7a-5p and that let-7a-5p targets TGFBR1, Smad2, and Col1a2. Rescue experiments in LX-2 cells further demonstrated that hsa_circ_0001377 promotes HSC activation, proliferation, migration, and G1/S-phase transition through the let-7a-5p/TGFBR1, Smad2, and Col1a2 axis. Moreover, hsa_circ_0001377 was highly expressed in 20 human HF samples, and its expression increased with advancing fibrosis stage. *In vivo*, a BDL-induced murine HF model demonstrated that the homologous mmu_circ_0001377 was also significantly upregulated in fibrotic liver tissues. In addition, tail-vein injection of an mmu-let-7a-5p agomir into BDL-induced HF mice confirmed the inhibitory effects of the let-7a-5p/TGFBR1, Smad2, and Col1a2 axis on α-SMA expression and collagen deposition.

**Conclusion**: Taken together, these findings suggest that the hsa_circ_0001377/let-7a-5p/TGFBR1, Smad2, and Col1a2 regulatory axis may represent a promising therapeutic target for HF.

## Graphical Abstract

**Figure:**
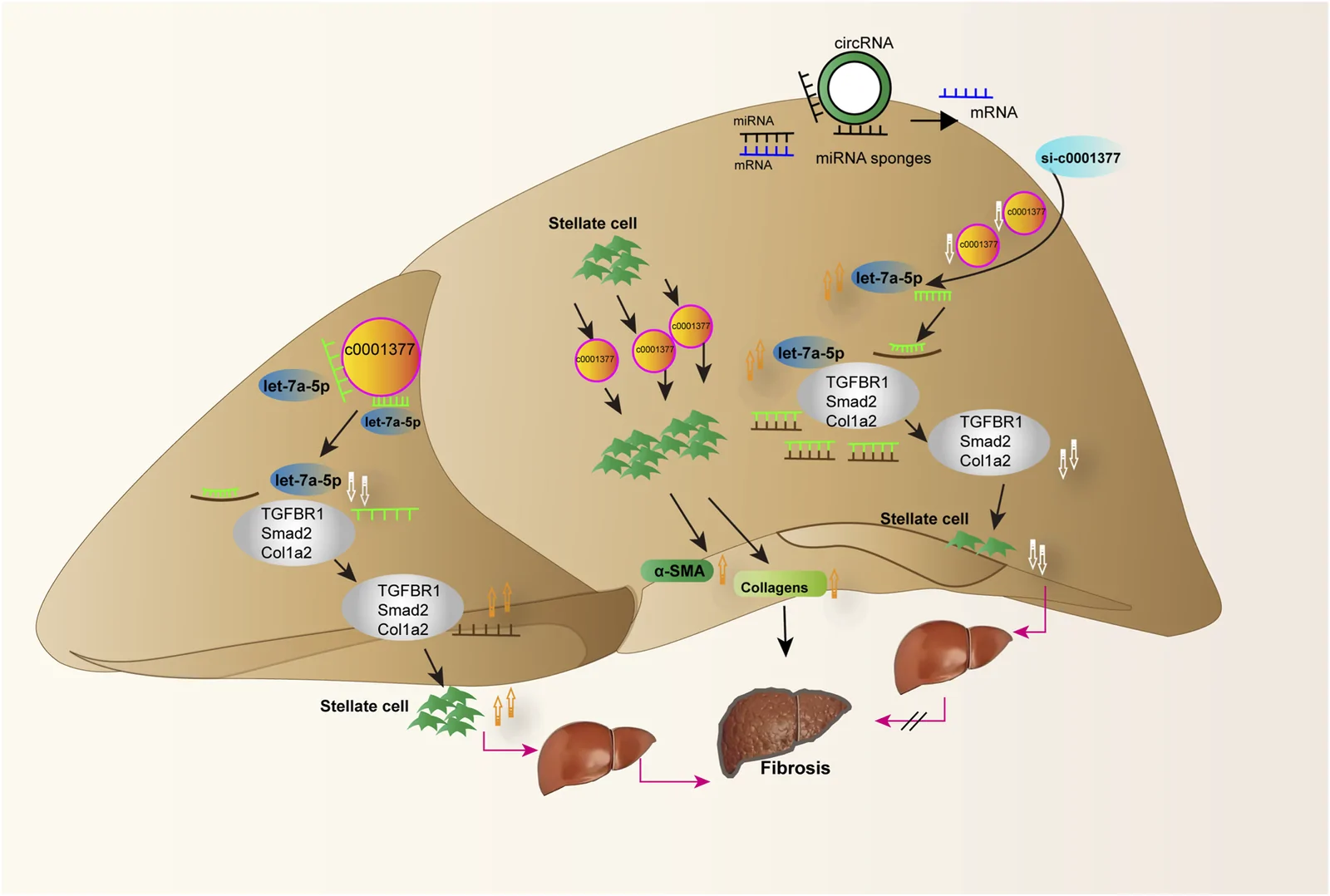
Diagram of how the hsa_circ_0001377/let-7a-5p/TGFBR1, Smad2, and Col1a2 pathway promotes liver fibrosis.

## Introduction

Hepatic fibrosis (HF), a major global health burden, is a complex reparative response to sustained damage in chronic liver diseases (Hernandez-Gea and Friedman, 2011). A central event in this process is the activation of hepatic stellate cells (HSCs) within the space of Disse (Higashi et al., 2017). Quiescent HSCs are rich in vitamin A lipid droplets. Following liver damage, HSCs lose their vitamin A stores and differentiate into activated myofibroblasts (MFs), which are characterized by the expression of α-smooth muscle actin (α-SMA). α-SMA expression is accompanied by morphological alterations in activated HSCs (aHSCs), which acquire enhanced proliferative, contractile, and migratory capabilities. During activation, aHSCs produce and deposit large amounts of extracellular matrix (ECM), predominantly comprising collagens, particularly collagen I, along with proteoglycans and glycoproteins. This process ultimately disrupts the physiological matrix within the space of Disse (Friedman, 2008; Zhang et al., 2016).

Without timely and effective intervention, HF may progress to cirrhosis and ultimately to fatal hepatocellular carcinoma (HCC). An estimated 1.5 billion individuals worldwide are affected by chronic liver disease, which causes approximately two million deaths annually; therefore, the population potentially requiring antifibrotic therapy is substantial (Massey et al., 2017; Wilson et al., 2017). However, no widely recognized effective therapeutic strategy for HF is currently available. Therefore, elucidating the molecular mechanisms underlying HF and identifying novel molecular targets for therapeutic intervention are urgently needed.

The regulatory roles of non-coding RNAs (ncRNAs) in organ fibrosis have attracted considerable attention in recent years (Abidan and Guo, 2021; Lu et al., 2021; Riaz and Li, 2019). Circular RNAs (circRNAs), characterized by a stable loop structure, have been identified as a novel class of ncRNAs (Chen et al., 2017). In 2013, two studies were concurrently published in the same issue of Nature, in which researchers identified numerous stably expressed circRNAs in humans, mice, and nematodes. These findings indicated that circRNAs exhibit cell type- or developmental stage-specific expression patterns (Sebastian et al., 2013; Thomas B et al., 2013). CircRNAs possess inherent biological stability, conservation, and tissue specificity, making them promising biological targets. Accumulating evidence has established a significant association between circRNAs and conditions such as myocardial fibrosis, idiopathic pulmonary fibrosis (IPF), and organ aging (Pan et al., 2022). In a renal fibrosis model using glucose-stressed HK-2 cells, downregulation of circACTR2 significantly reduced collagen IV and fibronectin synthesis (Si et al., 2020). However, research on circRNAs in HF remains in its infancy.

Our group collected six clinical HF samples for RNA sequencing to characterize the circRNA expression profiles in human HF tissues. Eight exon-derived circRNAs (ecircRNAs) with significant dysregulation were selected for initial validation. Among these circRNAs, hsa_circ_0001377 (circTNK2), derived from eight exons of the *TNK2* gene, was successfully amplified by qRT-PCR and showed the most pronounced upregulation in TGF-β1-aHSCs. Fluorescence *in situ* hybridization (FISH) and co-staining with α-SMA further demonstrated that hsa_circ_0001377 was abundantly expressed in aHSCs. Furthermore, hsa_circ_0001377 is highly conserved across species, and its murine homolog, mmu_circ_0001377 (http://www.circbank.cn/#/browse/details?type = mouse&id = mmu_Tnk2_0005000), was highly expressed in a mouse model of HF induced by bile duct ligation (BDL). Therefore, hsa_circ_0001377 may serve as a promising regulator of HF and was selected for further research.

## Materials and methods

### Patient samples

All liver tissue samples used in this study were obtained from the Second Affiliated Hospital of Naval Medical University, Shanghai, China. Each sample was reviewed independently by two professional pathologists. The stage of HF was determined using the METAVIR semi-quantitative scoring system. A total of 20 HF tissues (patient characteristics and fibrosis stages are presented in Supplementary Table S1) were used to assess hsa_circ_0001377 expression. Normal liver tissues were collected from patients undergoing surgical resection of hepatic hemangiomas. This study was approved by the Research Ethics Committee of the Second Affiliated Hospital of Naval Medical University, and written informed consent was obtained from all participants.

### Cell culture and TGF-β1 cytokine stimulation

The human hepatic stellate cell line LX-2 was cultured in DMEM supplemented with 100 U/mL penicillin, 100 mg/mL streptomycin, and 10% fetal bovine serum (Gibco, New York, NY, United States) at 37 °C in an atmosphere containing 5% CO_2_.

LX-2 cells in the logarithmic growth phase were seeded in culture dishes. After serum starvation in serum-free DMEM for 24 h, recombinant human TGF-β1 (15 ng/mL) was added to the cells. After 24 h of stimulation, the medium was replaced with DMEM containing 10% FBS, and the cells were cultured for an additional 24–48 h.

### CircRNA annotation and bioinformatics analysis

CircRNA annotation was performed using the circBank database and the UCSC Genome Browser. Potential miRNAs and their target genes were predicted using the TargetScan and DAVID databases. The murine homolog of hsa_circ_0001377 was obtained from the circBank database (http://www.circbank.cn/#/browse/details?type = mouse&id = mmu_Tnk2_0005000).

### Quantitative RT-PCR

Total RNA was extracted from LX-2 cells and patient tissues using TRIzol Reagent (Life Technologies, United States). After reverse transcription, an SYBR® Green Pro Taq HS PCR Kit (AG Technology, Shanghai, China) was used to detect circRNAs and messenger RNAs (mRNAs). The PCR conditions were as follows: 95 °C for 30 s, followed by 40 cycles of 95 °C for 5 s and 60 °C for 30 s. miRNA detection was performed using reverse transcription and qRT-PCR kits (RiboBio, Guangzhou, China). The PCR protocol was as follows: 95 °C for 10 min, followed by 40 cycles of 95 °C for 2 s, 60 °C for 20 s, and 70 °C for 10 s. mRNA and circRNA levels were normalized to GAPDH, while miRNA levels were normalized to U6.

The primer sequences for circRNAs and mRNAs are shown in Supplementary Tables S2 and S3. miRNA primer sequences were designed using patented technology developed by RiboBio (Guangzhou, China).

### Fluorescence *in situ* hybridization

The expression level of hsa_circ_0001377 in liver tissues was assessed using a Cy3-labeled hsa_circ_0001377 probe (circ103133, RiboBio, Guangzhou, China) and a fluorescence *in situ* hybridization kit (RiboBio, Guangzhou, China).

According to the manufacturer’s instructions, fibrotic liver sections were first incubated with prehybridization buffer at 37 °C for 30 min. Simultaneously, the hybridization solution was preheated to 37 °C, and a 20 μM FISH probe mixture was added under light-protected conditions. After removal of the prehybridization buffer, the probe-containing hybridization solution was added to the sections, which were then incubated at 37 °C overnight. The following day, the sections were sequentially washed with hybridization wash solutions I, II, and III at 42 °C for 5 min each in the dark, followed by rinsing with 1× PBS at room temperature for 5 min. DAPI solution was then added, and the sections were stained in the dark for 10 min. Fluorescence signals were examined using an oil-immersion fluorescence microscope (Olympus, Japan).

### RNase R treatment assay

The RNase R treatment assay was performed to evaluate the stability of hsa_circ_0001377. In brief, 1 μg of total RNA was incubated with RNase R or DEPC-treated water in a reaction system containing 10× reaction buffer (Epicentre Technologies, Madison, WI, United States) for 30 min at room temperature. For the RNase R group, 0.15 μL of RNase R (20 U/μL) and 1.5 μL of reaction buffer were added, while the control group received 0.15 μL of DEPC-treated water and 1.5 μL of reaction buffer. The digested RNA was subsequently reverse-transcribed and analyzed by qPCR. The qPCR products were also analyzed by agarose gel electrophoresis.

### Stable hsa_circ_0001377-overexpressing LX-2 cell line construction

The lentiviral vector GV689 (GeneChem, Shanghai, China) was used to construct the hsa_circ_0001377 overexpression vector, while the CON510 vector was employed as the negative control. The amplification primer sequences were as follows: forward primer (F), CTC​CCC​ACC​ATC​ACT​TTT​TAG​AGG​CTG​GGA​GGC​GGC​AGA​ATG, and reverse primer (R), AAC​TTG​GGA​AAT​TCT​TTT​GTA​CCT​TCC​CTC​GAT​GAC​GGT​GAT​GAC. The primers used for PCR identification were as follows: forward primer (F), CCT​CTA​CGG​GGT​GGT​GCT​CA, and reverse primer (R), ACA​TCA​CTT​TCC​CAG​TTT​ACC. Successful vector construction was confirmed by direct sequencing (Supplementary Figure S1). Stable hsa_circ_0001377-overexpressing and negative-control (NC) lentivirus-infected LX-2 cell lines were established through puromycin selection and verified by qRT-PCR.

### Design of small interfering RNAs targeting hsa_circ_0001377

To achieve hsa_circ_0001377 knockdown, three siRNAs targeting the back-splice junction of hsa_circ_0001377 were designed and chemically synthesized by Geneseed Biotech (Guangzhou, China) (Table 1).

**TABLE 1:** siRNA - hsa-circ_0001377 sequence design.

Gene	siRNA	Target seq
hsa_circ_0001377	siRNA1	UCAUCGAGGGAAGAGGCUGtt CAGCCUCUUCCCUCGAUGAtt
	siRNA2	CAUCGAGGGAAGAGGCUGGtt CCAGCCUCUUCCCUCGAUGtt
	siRNA3	AUCGAGGGAAGAGGCUGGGtt CCCAGCCUCUUCCCUCGAUtt

### Cell transfection

LX-2 cells in the logarithmic growth phase were seeded in culture dishes at an appropriate density. Plasmids and siRNAs were transfected into the corresponding experimental groups using Lipofectamine 3000 (Invitrogen, CA, United States) according to the manufacturer’s instructions, with the siRNAs used at a final concentration of 50 nM. miRNA products and transfection reagents were purchased from RiboBio (Guangzhou, China), and transfection was performed according to the manufacturer’s instructions at a final miRNA concentration of 125 nM.

### Western blot assay

At 48 h after transfection, LX-2 cells from each experimental group were lysed in RIPA buffer supplemented with PMSF at a ratio of 100:1. The proteins were subsequently separated on 10% SDS-polyacrylamide gels and transferred onto polyvinylidene fluoride membranes (Millipore, CA, United States) at 350 mA for 2 h. The membranes were incubated overnight at 4 °C with monoclonal primary antibodies against α-SMA, Col1a1, TGFBR1, Smad2, and Col1a2 (CST, Novus, United States). After incubation with the corresponding secondary antibodies (Novus, United States) for 2 h, protein bands were visualized using enhanced chemiluminescence (ECL) and imaged using a Tanon 3500 Imaging System (Tanon, Shanghai, China). GAPDH was used as the internal loading control.

### CCK-8 assay

At 48 h after transfection, LX-2 cells from each experimental group were collected, resuspended, and seeded into 96-well plates at a density of 1 × 10^3^ cells/mL. At 1, 2, 3, 4, and 5 days after seeding, 10 μL of CCK-8 reagent (Dojindo, Kumamoto, Japan) was added to each well, followed by incubation at room temperature for 4 h. Absorbance was measured at 450 nm using a microplate reader.

### Transwell migration assay

At 48 h after transfection, LX-2 cells from each experimental group were resuspended and seeded into the upper chambers of Transwell inserts (Corning, NY, United States) at a density of 1 × 10^6^ cells/mL. The cells were allowed to migrate for 48 h. Cells that had migrated through the membrane were stained with 1% crystal violet and counted in at least three high-magnification fields using an inverted microscope (Zeiss, Thornwood, United States).

### Cell cycle assay and apoptosis analysis

A cell cycle experiment was carried out using a cell cycle staining kit (Lianke Biotech, China). In brief, 1 × 10^6^ LX-2 cells from each experimental group were harvested and stained with PI (10 μL) for 30 min at 37 °C in the absence of light. Cell cycle was detected using a flow cytometer (Beckman Coulter, CA, United States) within 1 h.

Apoptosis was quantified using an Annexin V-APC Single Staining Kit (Lianke Biotech, China). Cells in each Falcon tube were incubated with 10 μL of Annexin V-APC at 37 °C in the dark for 10–15 min. Apoptosis was analyzed using a flow cytometer (Beckman Coulter, CA, United States) within 1 h of staining.

### AGO-RIP assay

An RNA Immunoprecipitation Kit (Geneseed Biotech, Guangzhou, China) was used for RNA immunoprecipitation (RIP). LX-2 cells (1 × 10^7^) were pelleted and resuspended in an equal volume of RIP lysis buffer. Cell lysates (100 μL) were incubated overnight at 4 °C with 5 μg of an anti-Argonaute-2 (AGO2) antibody (CST, MA, United States) or isotype-matched IgG-coated beads (CST, MA, United States). Following proteinase K treatment, the immunoprecipitated RNAs were extracted according to the manufacturer’s protocol. The abundance of hsa_circ_0001377 and let-7a-5p was determined by qRT-PCR.

### Luciferase reporter assay

To assess the binding between hsa_circ_0001377 and let-7a-5p, wild-type (WT) or mutant (MT) hsa_circ_0001377 sequences were inserted into the psiCHECK™ vector. HEK293T cells were cotransfected with either the let-7a-5p mimic or mimic NC and the hsa_circ_0001377-WT-psiCHECK™ or hsa_circ_0001377-MT-psiCHECK™ plasmid using Lipofectamine 3000 (Invitrogen, CA, United States). At 48 h after transfection, relative luciferase activity was measured using the Dual-Luciferase Reporter Assay System (Promega, Madison, WI, United States) and normalized to firefly luciferase activity.

Additionally, the WT or corresponding MT 3′ untranslated region (3′ UTR) sequences of TGFBR1, Smad2, and Col1a2 were inserted into the psiCHECK™ vector. The let-7a-5p mimic or mimic NC was cotransfected with the corresponding 3′ UTR-WT-psiCHECK™ or 3′ UTR-MT-psiCHECK™ vector of TGFBR1, Smad2, or Col1a2. At 48 h after transfection, the relative luciferase activity in each group was measured using the Dual-Luciferase Reporter Assay System (Promega, Madison, WI, United States).

### miRNA pulldown assay

LX-2 cells (1 × 10^7^) were harvested and lysed for the miRNA pulldown assay. A biotinylated let-7a-5p probe (ssR220105013314) or a biotinylated negative-control (NC) miRNA probe was incubated with C-1 magnetic beads (Life Technologies, Guilford, CT, United States) at 25 °C for 2 h. The cell lysates were then incubated with the probe-coated beads at 4 °C overnight. The following day, the bead-bound RNA complexes were eluted and extracted for qRT-PCR analysis. All biotinylated miRNA probes were designed and synthesized by RiboBio (Guangzhou, China).

### BDL mouse HF model construction

Female C57BL/6 mice aged 4–6 weeks were purchased from the Chinese Academy of Sciences (Shanghai, China). The mice were housed under specific pathogen-free conditions, and all experimental procedures were approved by the Research Ethics Committee of the Second Affiliated Hospital of Naval Medical University, Shanghai, China. To assess mmu_circ_0001377 expression *in vivo*, the mice were randomly assigned to two groups: a control group without surgical intervention (n = 3) and a BDL group (n = 12). In brief, mice undergoing BDL were anesthetized with halothane, and a midline laparotomy was performed. The extrahepatic common bile duct was double-ligated with 6–0 silk sutures and transected between the ligatures. The abdominal incision was then closed with silk sutures, and the mice were allowed to recover in separate cages. Four weeks after surgery, the mice were euthanized.

Two additional experimental groups were established to receive tail-vein injections of either mmu-let-7a-5p agomir or agomir negative control (agomir NC). In brief, 7 days after BDL surgery, mice in the BDL + mmu-let-7a-5p agomir group (n = 10) and BDL + agomir NC group (n = 10) received tail-vein injections of mmu-let-7a-5p agomir or agomir NC, respectively. Twenty nanomoles of each agomir were dissolved in 200 μL of saline and administered twice weekly. All BDL mice were euthanized after receiving six injections. The mmu-let-7a-5p agomir and agomir NC were purchased from RiboBio (Guangzhou, China).

Liver tissues were collected and embedded in paraffin for hematoxylin and eosin (H&E), Masson’s trichrome, and immunohistochemical (IHC) staining. A portion of each tissue sample was stored at −80 °C for RNA extraction and qRT-PCR analysis.

### H&E, Masson, and IHC staining

Harvested mouse liver tissues were fixed in 10% formalin and embedded in paraffin, after which serial sections with a thickness of 4 μm were prepared. Routine H&E and Masson’s trichrome staining were performed for histological assessment. For IHC staining, the sections were routinely deparaffinized and rehydrated. The sections were subsequently incubated with antibodies against α-SMA, Col1a1, TGFBR1, Smad2, and Col1a2 (CST, Novus, United States) at room temperature for 30 min and then maintained at 4 °C overnight. The following day, immunoreactivity was visualized using diaminobenzidine, followed by hematoxylin counterstaining. Images were captured using a microscope (Zeiss, Thornwood, United States). Masson-positive and IHC-positive areas were quantified using ImageJ software by analyzing at least five randomly selected fields per section. Scoring was performed by observers blinded to the treatment groups.

### Statistical analysis

Statistical analyses were performed using SPSS version 25.0 and GraphPad Prism version 9.0. Data normality and homogeneity of variance were assessed using the Shapiro-Wilk test and F test before parametric tests were applied. Between-group differences were analyzed using an unpaired Student’s t-test, Welch’s t-test, Mann–Whitney U test, or Wilcoxon matched-pairs signed-rank test, as appropriate. Data are expressed as the mean ± standard deviation (SD) from three independent experiments. A *p-*value <0.05 was considered statistically significant. Asterisks indicate statistical significance as follows: *p < 0.05, **p < 0.01, and ***p < 0.001.

## Results

### Identification and characterization of hsa_circ_0001377 (circTNK2) in HF tissues and HSCs

Bioinformatics analysis was performed using six clinical HF samples previously collected and sequenced by our group (Binbin et al., 2022). Based on gene composition, expression abundance, and sequence length (500–1,500 bp), eight significantly upregulated exonic circRNAs (fold change >2, *p* < 0.05) were initially selected for qRT-PCR analysis (Figure 1A; Supplementary Table S4). Divergent primers were designed (Supplementary Table S2), and qRT-PCR analysis revealed single peaks in the melting curves of circCUX2, circFGF2, circD2HGDH, circTNK2, and circPER3, confirming successful amplification of these five circRNAs in the human HSC line LX-2 (Supplementary Figure S2). LX-2 cells were subsequently stimulated with the potent profibrogenic factor TGF-β1 (15 ng/mL) (Katsunori and Koichi, 2012). qRT-PCR analysis showed that circTNK2 exhibited the greatest increase following TGF-β1 stimulation, accompanied by increased expression of the HF markers α-SMA and Col1a1 (Figure 1B).

**FIGURE 1:**
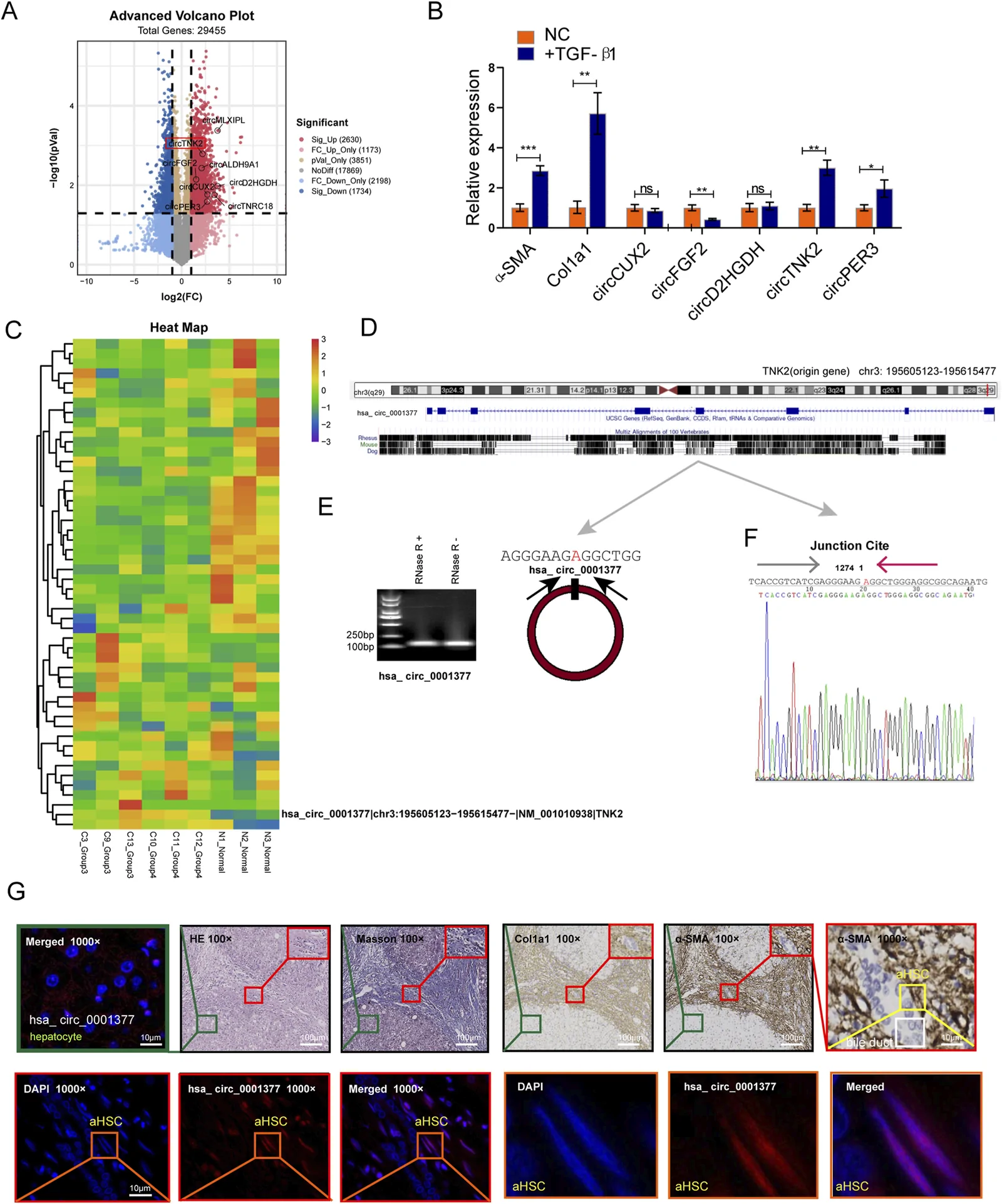
Identification and characteristics of hsa_circ_0001377 (circTNK2) in HF tissues and HSCs. **(A)** Volcano plot of differentially expressed circRNAs. The x-axis represents log_2_ (fold change), and the y-axis represents −log_10_(P value). **(B)** qRT-PCR analysis of circRNA expression in TGF-β1-activated LX-2 cells. **(C)** Clustered heatmap of differentially expressed circRNAs. Rows represent circRNAs, and columns represent HF samples. **(D)** Schematic illustration showing that hsa_circ_0001377 is generated through the back-splicing of exons 2–9 of the *TNK2* gene. **(E)** Agarose gel electrophoresis showing that hsa_circ_0001377 was resistant to RNase R digestion. **(F)** The back-splice junction of hsa_circ_0001377 was verified by Sanger sequencing. **(G)** FISH analysis of HF tissues. Fibrotic areas were identified by H&E, Masson’s trichrome, and Col1a1 staining (100×). aHSCs were identified by staining for the specific marker α-SMA (100× and 1,000×). FISH showed that hsa_circ_0001377 was predominantly localized in the cytoplasm of aHSCs (1,000×). Cy3-labeled hsa_circ_0001377 is shown in red, and nuclei are shown in blue. RNA expression levels were normalized to GAPDH. Data were obtained from three independent experiments. Data normality and homogeneity of variance were assessed using the Shapiro–Wilk test and F test. Statistical significance was determined using an unpaired Student’s t-test. Error bars represent the standard deviation.

The circBank database was used for gene annotation, confirming that circTNK2 identified in our sequencing data corresponded precisely to hsa_circ_0001377 documented in circBank (http://www.circbank.cn/#/browse/details?type=human&id=hsa_TNK2_0001200). Analysis using the UCSC Genome Browser revealed that hsa_circ_0001377 is located at chr3:195605123–195615477 and is generated through the back-splicing of eight exons of the tyrosine kinase non-receptor 2 (*TNK2*) gene (Figures 1C,D). RNase R digestion assays demonstrated that hsa_circ_0001377 was highly resistant to RNase R digestion (Figure 1E; Supplementary Figure S3). The back-splice junction of hsa_circ_0001377 was further confirmed by Sanger sequencing (Figure 1F). FISH using a Cy3-labeled hsa_circ_0001377 probe, combined with α-SMA co-staining, revealed abundant cytoplasmic expression of hsa_circ_0001377 in aHSCs (α-SMA-positive) within the fibrous septa of HF tissues (Figure 1G). Collectively, these findings confirm the circular structure of hsa_circ_0001377 and suggest that it may serve as a key regulator of HF.

### Overexpression of hsa_circ_0001377 promotes LX-2 cell activation, proliferation, migration, and G1/S-phase transition

A GFP-labeled hsa_circ_0001377-overexpressing lentiviral vector (OV-hsa-circ_0001377-GV689) was successfully constructed (Figure 2A; Supplementary Figure S1). Viral titer analysis showed a concentration of 1 × 10^9^ TU/mL, which was sufficient for subsequent experiments (Supplementary Figure S4). LX-2 cells were then transduced with OV-hsa-circ_0001377-GV689 or NC lentivirus (Figure 2A). qRT-PCR analysis revealed that hsa_circ_0001377 expression was increased by 132-fold in LX-2 cells transduced with OV-hsa-circ_0001377-GV689 compared to those transduced with NC lentivirus (Figure 2A).

**FIGURE 2:**
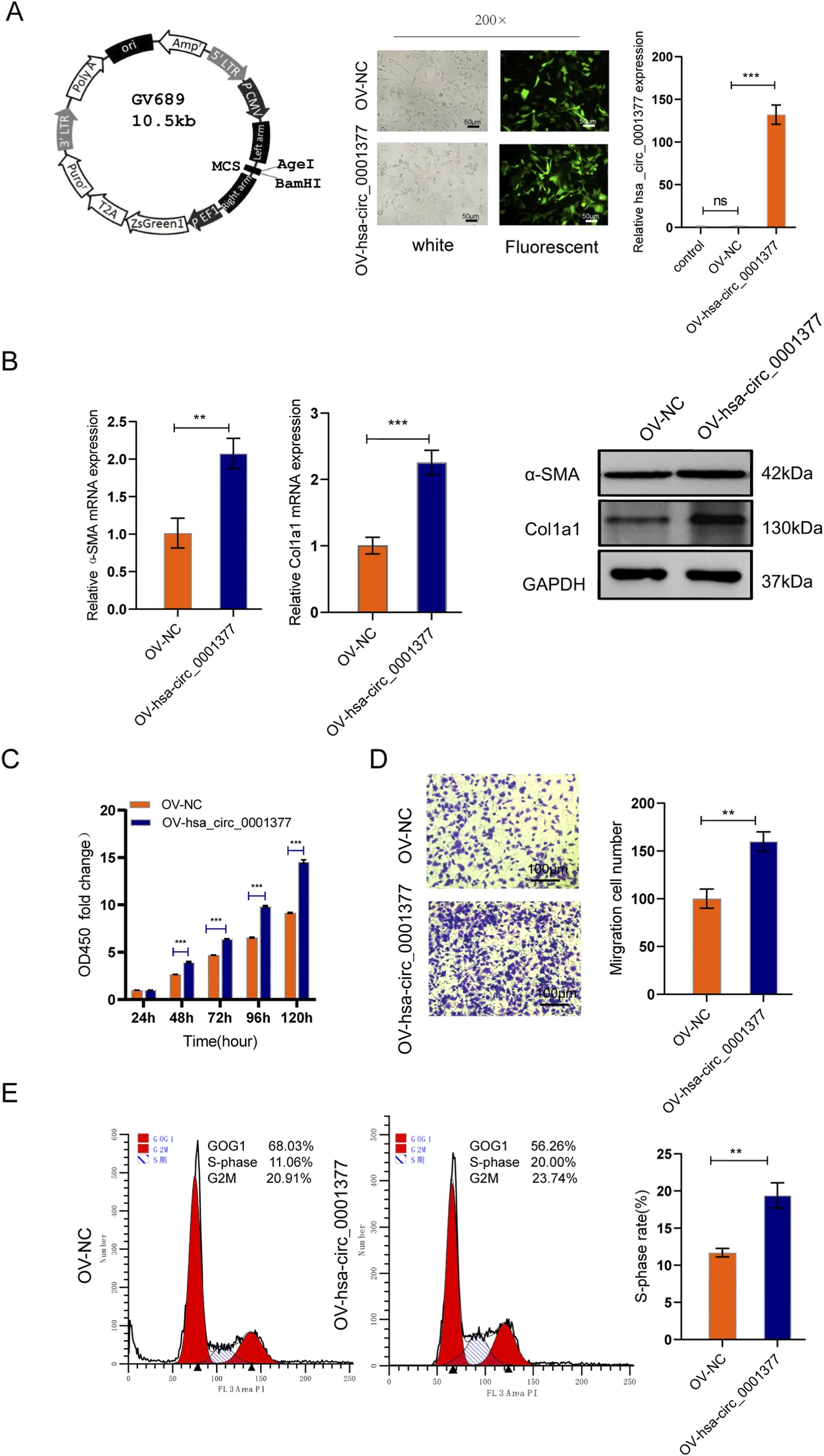
Overexpression of hsa_circ_0001377 promotes the activation, proliferation, migration, and G1/S transition of LX-2 cells. **(A)** GFP-labeled GV689 lentiviral vector was used to construct an hsa_circ_0001377-overexpressing lentivirus, which was subsequently used to infect LX-2 cells (magnification, ×200). hsa_circ_0001377 expression was assessed in the OV-NC and OV-hsa_circ_0001377 groups. qRT-PCR analysis confirmed the successful establishment of a stable hsa_circ_0001377-overexpressing LX-2 cell line. **(B)** mRNA and protein expression levels of α-SMA and Col1a1 in the OV-NC and OV-hsa_circ_0001377 groups. **(C–E)** CCK-8, Transwell migration, and cell cycle assays evaluating the proliferation, migration, and G1/S-phase transition of LX-2 cells in the OV-NC and OV-hsa_circ_0001377 groups. RNA and protein expression levels were normalized to GAPDH. Data were obtained from three independent experiments. Data normality and homogeneity of variance were assessed using the Shapiro–Wilk test and F test. Statistical significance was determined using an unpaired Student’s t-test or Welch’s t-test, as appropriate. Error bars represent the standard deviation.

As shown in Figure 2B, hsa_circ_0001377 overexpression markedly increased the expression of α-SMA and Col1a1. Furthermore, CCK-8, Transwell migration, and cell cycle assays demonstrated that hsa_circ_0001377 overexpression significantly enhanced LX-2 cell proliferation, migration, and G1/S phase transition (Figures 2C–E). These findings suggest that hsa_circ_0001377 functions as a potent profibrotic regulator in HF.

### Knockdown of hsa_circ_0001377 attenuates LX-2 cell activation, proliferation, and migration while promoting apoptosis

Three siRNAs targeting the back-splice junction of hsa_circ_0001377 were designed to silence its expression (Table 1; Figure 3A). qRT-PCR analysis demonstrated that hsa_circ_0001377 expression was significantly reduced in LX-2 cells transfected with si1-hsa_circ_0001377, si2-hsa_circ_0001377, or si3-hsa_circ_0001377 compared with cells transfected with negative-control siRNA (si-NC). si2-hsa_circ_0001377, hereafter referred to as si-hsa_circ_0001377, produced the greatest knockdown efficiency and was therefore selected for subsequent experiments (Figure 3A). However, si-hsa_circ_0001377 did not affect the expression of linear TNK2 mRNA (Supplementary Figure S5).

**FIGURE 3:**
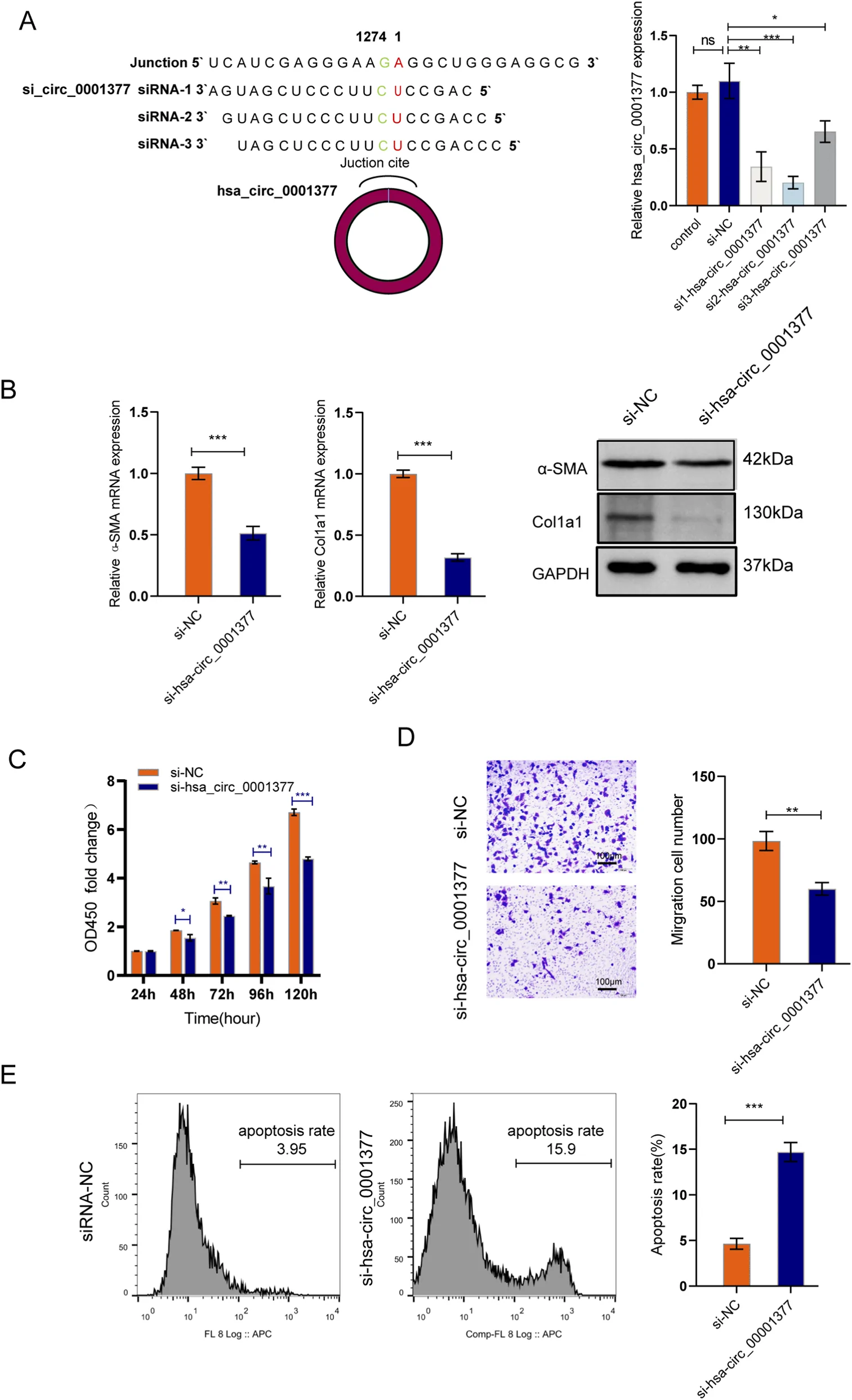
Knockdown of hsa_circ_0001377 inhibits the activation, proliferation, and migration of LX-2 cells. **(A)** Three siRNAs targeting the back-splice junction of hsa_circ_0001377 were designed. qRT-PCR was used to measure hsa_circ_0001377 expression in the si-NC and si-hsa_circ_0001377 groups. **(B)** mRNA and protein expression levels of α-SMA and Col1a1 in the si-NC and si-hsa_circ_0001377 groups. **(C–E)** CCK-8, Transwell migration, and Annexin V-APC assays evaluating the proliferation, migration, and apoptosis of LX-2 cells in the si-NC and si-hsa_circ_0001377 groups. RNA and protein expression levels were normalized to GAPDH. Data were obtained from three independent experiments. Data normality and homogeneity of variance were assessed using the Shapiro–Wilk test and F test. Statistical significance was determined using an unpaired Student’s t-test or Welch’s t-test, as appropriate. Error bars represent the standard deviation.

Knockdown of hsa_circ_0001377 significantly reduced the expression of the HF markers α-SMA and Col1a1 (Figure 3B). Moreover, CCK-8 and Transwell migration assays revealed decreases in cell proliferation and migration, while Annexin V-APC staining indicated increased apoptosis (Figures 3C–E). These results indicate that hsa_circ_0001377 knockdown suppresses HSC activation, proliferation, and migration while promoting apoptosis, suggesting that hsa_circ_0001377 may represent a potential therapeutic target for HF.

### Hsa_circ_0001377 directly targets let-7a-5p

The miRanda algorithm was utilized to predict potential miRNA-binding sites within hsa_circ_0001377, revealing that four members of the let-7 family had the highest binding scores (Figure 4A; Supplementary Table S5). The let-7 family was significantly downregulated in our miRNA-sequencing data from the same cohort of HF specimens (https://www.ncbi.nlm.nih.gov/geo/query/acc.cgi?acc=GSE190366) (Figure 4B). qRT-PCR and CCK-8 assays were initially performed to evaluate the functions of let-7 family members, including let-7a-5p, let-7c-5p, let-7f-5p, and let-7g-5p. The expression of both α-SMA and Col1a1 was significantly reduced in LX-2 cells transfected with let-7 mimics compared with the NC group, with the greatest reduction observed in cells transfected with the let-7a-5p mimic (Supplementary Figure S6). CCK-8 assays showed that LX-2 cell proliferation was reduced in all let-7 mimics-transfected groups compared with the NC group, with let-7a-5p exerting the strongest inhibitory effect (Supplementary Figure S7). These findings suggest that members of the let-7 family may function as negative regulators of HF. Given its particularly strong regulatory activity, let-7a-5p was selected for subsequent validation studies.

**FIGURE 4:**
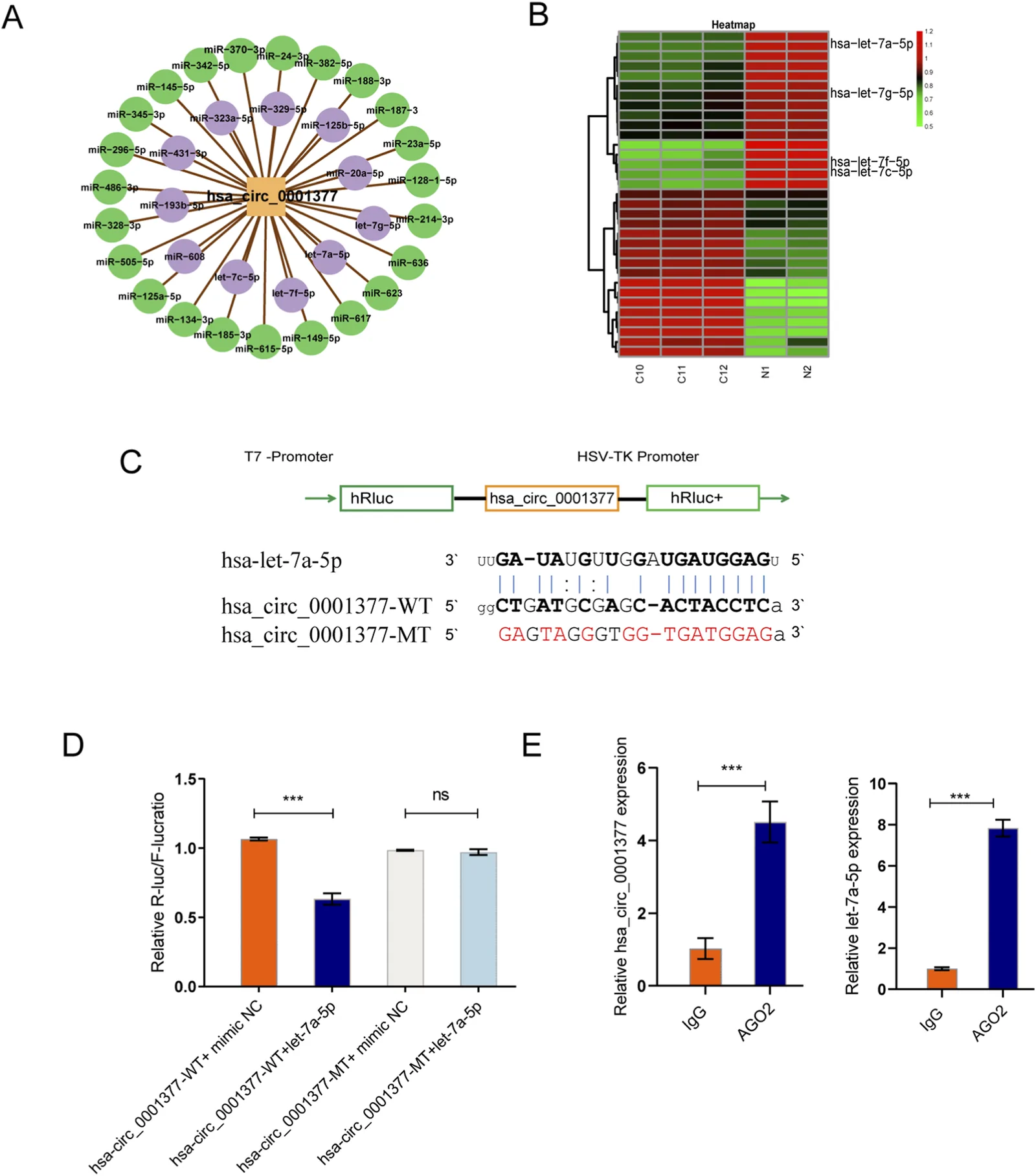
hsa_circ_0001377 directly targets let-7a-5p. **(A)** Bioinformatic prediction of miRNAs potentially binding to hsa_circ_0001377. **(B)** Clustered heatmap showing that let-7a-5p, let-7c-5p, let-7f-5p, and let-7g-5p were significantly downregulated in the same cohort of sequenced HF samples. **(C,D)** Predicted binding sites between hsa_circ_0001377 and let-7a-5p and validation of their interaction using a dual-luciferase reporter assay. **(E)** hsa_circ_0001377 and let-7a-5p were significantly enriched in AGO2 immunoprecipitates. RNA and miRNA expression levels were normalized to GAPDH and U6, respectively. Data were obtained from three independent experiments. Data normality and homogeneity of variance were assessed using the Shapiro–Wilk test and F test. Statistical significance was determined using an unpaired Student’s t-test. Error bars represent the standard deviation.

A luciferase reporter assay was conducted to validate the interaction between let-7a-5p and hsa_circ_0001377 using WT and mutant (MT) hsa_circ_0001377-psiCHECK™ plasmids (Figure 4C). As shown in Figure 4D, cotransfection of LX-2 cells with the let-7a-5p mimic and hsa_circ_0001377-WT plasmid significantly reduced luciferase activity compared with cotransfection with the mimic NC and the WT plasmid. In contrast, luciferase activity did not differ significantly between LX-2 cells cotransfected with the let-7a-5p mimic and hsa_circ_0001377-MT plasmid and those cotransfected with mimic NC and the MT plasmid. Subsequent AGO2-RIP assays demonstrated significant enrichment of both hsa_circ_0001377 and let-7a-5p in AGO2 immunoprecipitates, further supporting their interaction (Figure 4E). These results confirm that hsa_circ_0001377 directly binds let-7a-5p.

### Let-7a-5p functions as an antifibrotic regulator by directly targeting TGFBR1, Smad2, and Col1a2

Stimulation of LX-2 cells with the potent profibrogenic factor TGF-β1 (15 ng/mL) markedly reduced let-7a-5p expression (Figure 5A). The antifibrotic effects of let-7a-5p were subsequently validated using qRT-PCR (Figure 5B), Western blotting (Figure 5B), CCK-8, Transwell migration, and Annexin V-APC apoptosis (Figures 5C–E) assays, which demonstrated that let-7a-5p inhibited LX-2 cell activation, proliferation, and migration while promoting apoptosis.

**FIGURE 5:**
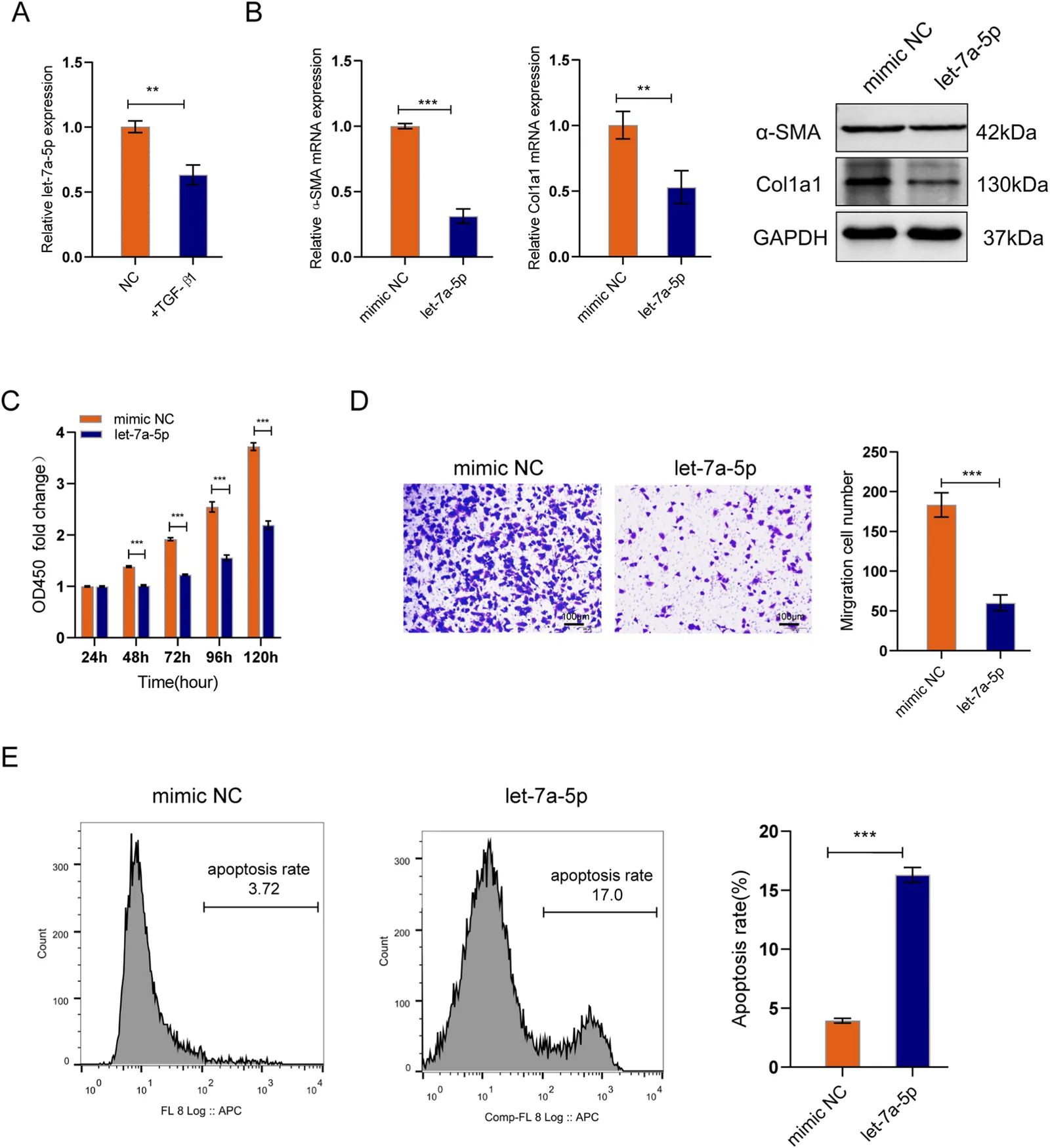
Let-7a-5p functions as an antifibrotic regulator. **(A)** qRT-PCR analysis showing significant downregulation of let-7a-5p in TGF-β1-activated LX-2 cells. **(B)** qRT-PCR and Western blot analyses of α-SMA and Col1a1 expression in the mimic NC and let-7a-5p mimic groups. **(C–E)** CCK-8, Transwell migration, and Annexin V-APC assays evaluating the proliferation, migration, and apoptosis of LX-2 cells in the mimic NC and let-7a-5p mimic groups. miRNA expression levels were normalized to U6, whereas RNA and protein expression levels were normalized to GAPDH. Data were obtained from three independent experiments. Data normality and homogeneity of variance were assessed using the Shapiro–Wilk test and F test. Statistical significance was determined using an unpaired Student’s t-test. Error bars represent the standard deviation.

Gene Ontology enrichment analysis revealed that the predicted downstream targets of the let-7 family were significantly enriched in the cytoplasm and cytosol and were closely associated with protein binding, kinase activity, receptor activity, and signal transduction (Figure 6A). Integrated analysis using the TargetScan and DAVID databases identified several HF-related genes as potential downstream targets of let-7a-5p (http://www.targetscan.org/vert_71/) (Figure 6B). Primers were specifically designed for these candidate genes (Supplementary Table S3), and qRT-PCR analysis showed that TGFBR1, Smad2, and Col1a2 exhibited the greatest reductions in expression following transfection of LX-2 cells with the let-7a-5p mimic (Figure 6C). Furthermore, a biotinylated let-7a-5p pulldown assay demonstrated significant enrichment of TGFBR1, Smad2, and Col1a2 transcripts in the let-7a-5p-bound fraction compared with the miRNA NC probe group (Figure 6D). Dual-luciferase reporter assays further confirmed the specific binding of let-7a-5p to TGFBR1, Smad2, and Col1a2 (Figures 6E,F). These results suggest that TGFBR1, Smad2, and Col1a2 are direct targets of let-7a-5p.

**FIGURE 6:**
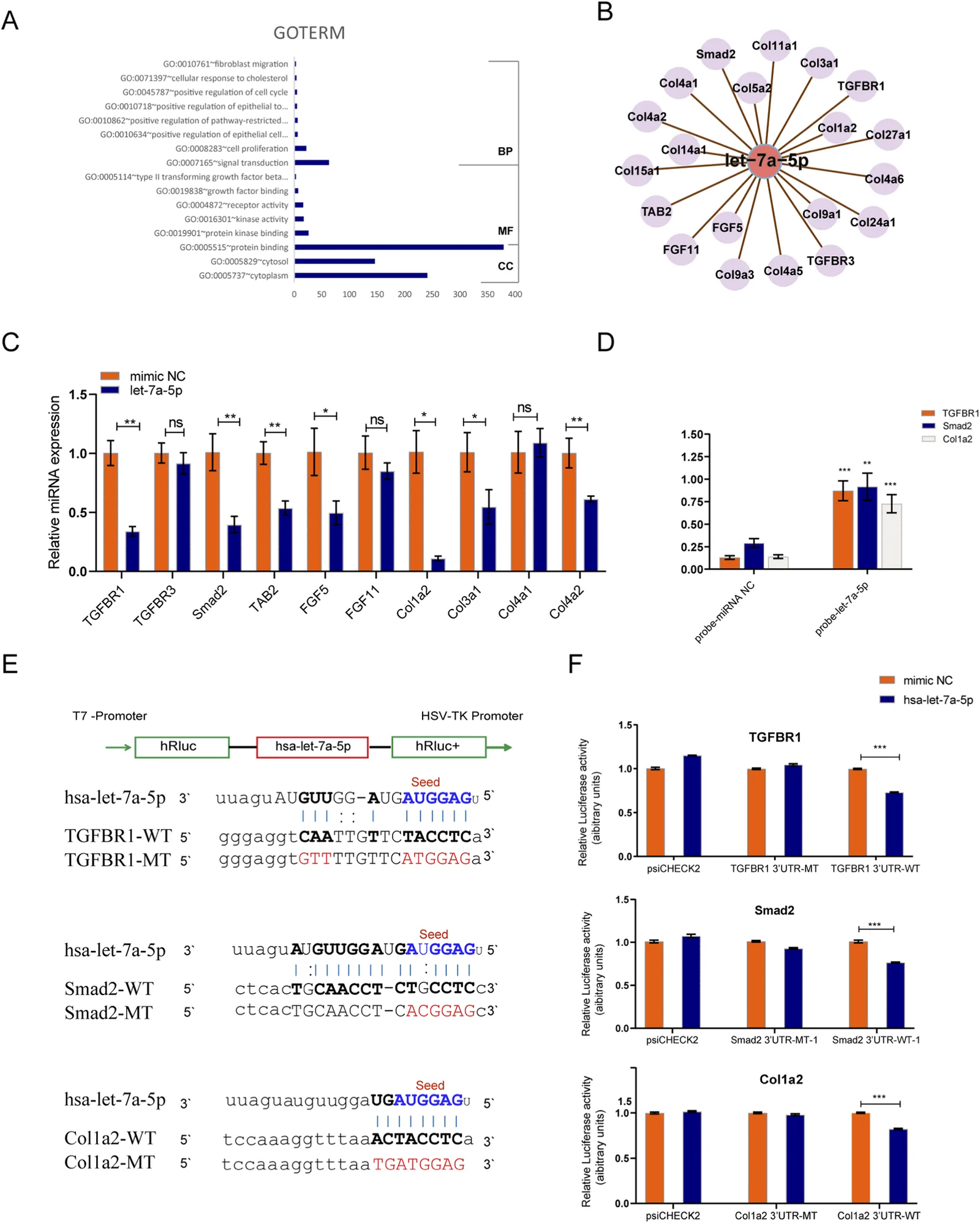
Let-7a-5p directly targets TGFBR1, Smad2, and Col1a2. **(A)** Gene Ontology enrichment analysis of the predicted downstream target genes of the let-7 family. **(B)** Network diagram illustrating the predicted mRNA targets of let-7a-5p. **(C)** Initial screening of potential let-7a-5p target genes by qRT-PCR. **(D)** miRNA pulldown assays confirming the interactions between let-7a-5p and TGFBR1, Smad2, and Col1a2. **(E,F)** Dual-luciferase reporter assays confirming the direct binding of let-7a-5p to TGFBR1, Smad2, and Col1a2. RNA expression levels were normalized to GAPDH. Data were obtained from three independent experiments. Data normality and homogeneity of variance were assessed using the Shapiro–Wilk test and F test. Statistical significance was determined using an unpaired Student’s t-test or Welch’s t-test, as appropriate. Error bars represent the standard deviation.

### Let-7a-5p attenuates the profibrotic effects of hsa_circ_0001377 by regulating TGFBR1, Smad2, and Col1a2

We hypothesized that the abundant hsa_circ_0001377 expressed in stable OV-hsa-circ_0001377 LX-2 cells acts as a sponge for let-7a-5p, thereby impairing its ability to suppress downstream target genes. As shown in Figure 7A, the expression levels of TGFBR1, Smad2, and Col1a2 were significantly higher in OV-hsa-circ_0001377 LX-2 cells than in OV-NC cells, supporting our hypothesis that hsa_circ_0001377 sequesters let-7a-5p and thereby increases the expression of its downstream targets. Furthermore, transfection with the let-7a-5p mimic significantly reversed the elevated expression of TGFBR1, Smad2, and Col1a2 in stable OV-hsa-circ_0001377 LX-2 cells, confirming that hsa_circ_0001377 regulates these genes through its interaction with let-7a-5p (Figure 7A). In addition, the let-7a-5p mimic partially attenuated the increased expression of α-SMA and Col1a1 (Figure 7B) and inhibited the proliferation, migration, and G1/S-phase transition (Figures 7C–E) of stable OV-hsa-circ_0001377 LX-2 cells. Collectively, these findings indicate that hsa_circ_0001377 promotes HF by competitively binding to let-7a-5p and modulating the expression of TGFBR1, Smad2, and Col1a2.

**FIGURE 7:**
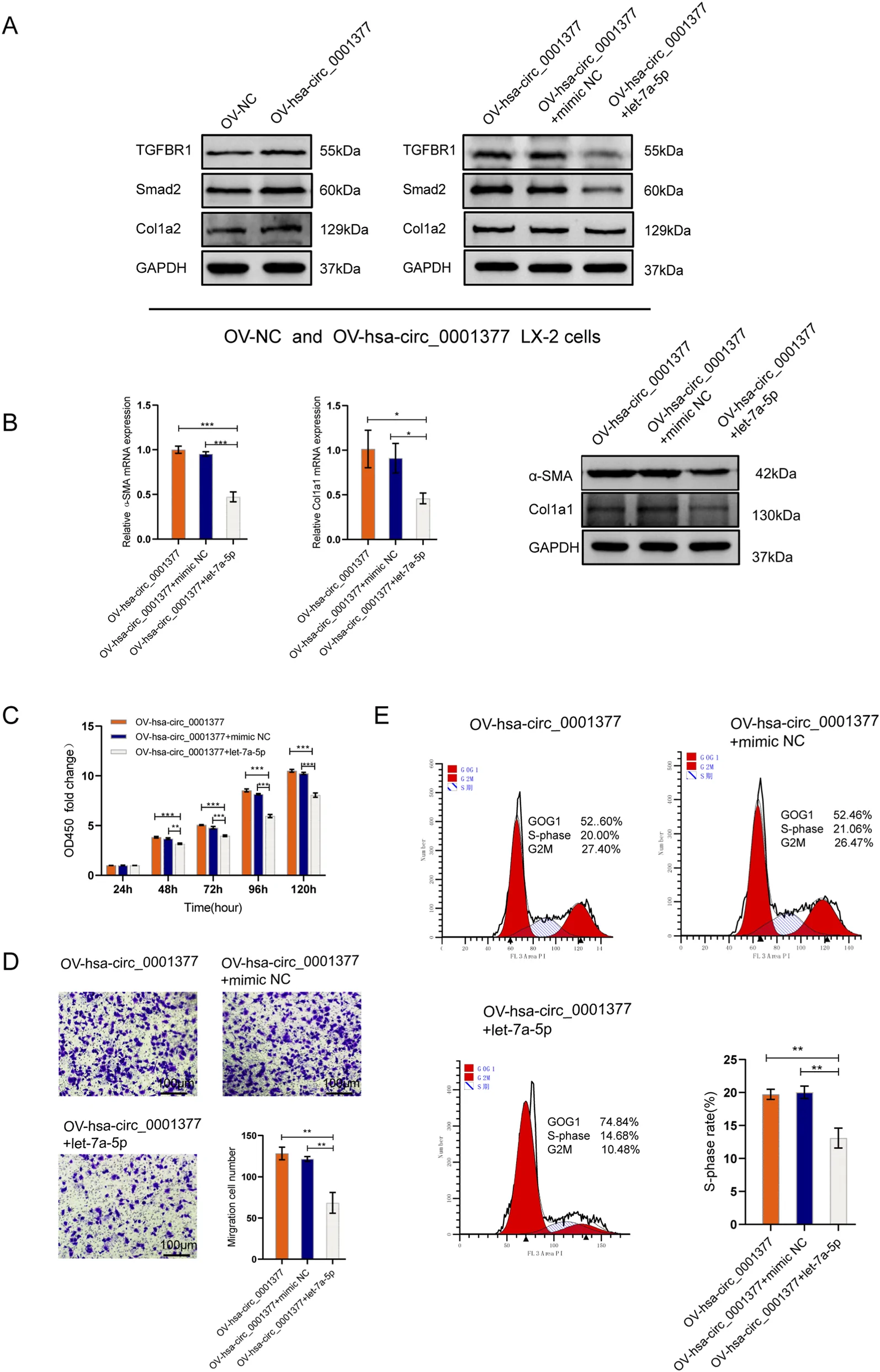
Let-7a-5p eliminates the pro-fibrotic effect of hsa_circ_0001377 *via* regulating TGFBR1, Smad2, and Col1a2. **(A)** Protein expression levels of TGFBR1, Smad2, and Col1a2 in each experimental group. **(B)** mRNA and protein expression levels of α-SMA and Col1a1 in each experimental group. **(C,D,E)** CCK-8, Transwell migration, and cell cycle assays evaluating the proliferation, migration, and G1/S-phase transition of LX-2 cells in each experimental group. RNA and protein expression levels were normalized to GAPDH. Data were obtained from three independent experiments. Data normality and homogeneity of variance were assessed using the Shapiro–Wilk test and F test. Statistical significance was determined using an unpaired Student’s t-test. Error bars represent the standard deviation.

### hsa_circ_0001377 expression is positively associated with clinical HF stage, and let-7a-5p attenuates HF progression in BDL mice

A total of 20 human HF tissue samples were collected, with fibrosis stages distributed as follows: F1, 2/20 (10%); F2, 4/20 (20%); F3, 5/20 (25%); and F4, 9/20 (45%) (Figure 8A; Supplementary Table S1). qRT-PCR analysis confirmed that hsa_circ_0001377 expression was higher in HF tissues than in matched normal liver tissues (Figure 8B). Furthermore, hsa_circ_0001377 expression was markedly higher in F3-F4 tissues than in F1-F2 tissues (Figure 8B). Additionally, qRT-PCR and IHC analyses revealed that samples with elevated hsa_circ_0001377 expression exhibited concomitantly reduced let-7a-5p expression and markedly increased TGFBR1, Smad2, and Col1a2 expression (Figure 8C). In parallel, a mouse model of HF was established by common BDL (Figure 8D). Four weeks after BDL, the mice were euthanized, and total RNA was extracted from liver tissues for qRT-PCR analysis. The results showed that mmu_circ_0001377 expression was significantly increased in the BDL group compared with the control group, accompanied by increased expression of the HF markers α-SMA and Col1a1 (Figure 8E). Consistently, qRT-PCR and IHC analyses revealed that elevated mmu_circ_0001377 expression was associated with lower mmu-let-7a-5p expression and higher TGFBR1, Smad2, and Col1a2 expression (Figure 8F). These findings demonstrate that hsa_circ_0001377 and its murine homolog, mmu_circ_0001377, exhibit similar expression patterns across species, highlighting the potential of hsa_circ_0001377 as a novel and sensitive biomarker for HF.

**FIGURE 8:**
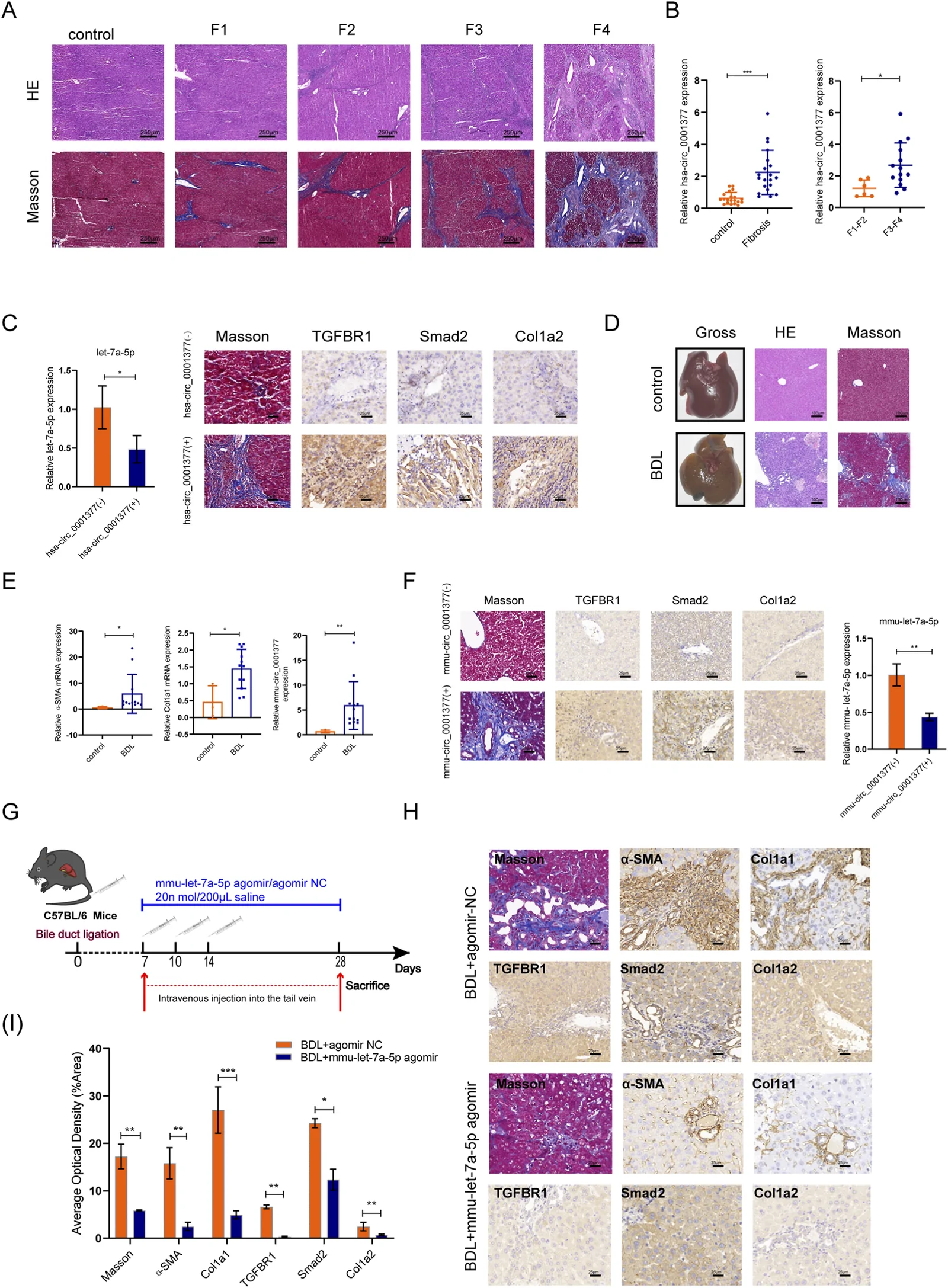
Hsa_circ_0001377 is positively correlated with clinical HF staging, and let-7a-5p/TGFBR1, Smad2, Col1a2 axis attenuates HF progression in BDL mice **(A)** H&E and Masson’s trichrome staining of human HF tissues. **(B)** qRT-PCR analysis showing that hsa_circ_0001377 was significantly upregulated in liver tissues from 20 patients with HF. Furthermore, hsa_circ_0001377 expression was higher in patients with F3-F4 fibrosis than in those with F1-F2 fibrosis. **(C)** Expression of let-7a-5p, TGFBR1, Smad2, and Col1a2 in human HF tissues. **(D)** Gross morphology and H&E and Masson’s trichrome staining of liver tissues from BDL mice. **(E)** qRT-PCR analysis showing that mmu_circ_0001377, α-SMA, and Col1a1 were highly expressed in fibrotic liver tissues from BDL mice. **(F)** Expression of mmu-let-7a-5p, TGFBR1, Smad2, and Col1a2 in fibrotic liver tissues from BDL mice. **(G,H,I)** Tail-vein administration of mmu-let-7a-5p agomir to BDL-induced fibrotic mice suppressed TGFBR1, Smad2, and Col1a2 expression, thereby reducing α-SMA and Col1a1 levels. Data were obtained from three independent experiments. Data normality and homogeneity of variance were assessed using the Shapiro–Wilk test and F test. Statistical significance was determined using the Wilcoxon matched-pairs signed-rank test **(B)**, unpaired Student’s t-test **(C,F,I)**, or Mann–Whitney U test **(E)**, as appropriate. Error bars represent the standard deviation.

Furthermore, Masson’s trichrome staining revealed significantly reduced collagen fiber deposition in the BDL + mmu-let-7a-5p agomir group compared with the BDL + agomir NC group. IHC staining also demonstrated significantly lower expression of α-SMA, Col1a1, TGFBR1, Smad2, and Col1a2 in the BDL + mmu-let-7a-5p agomir group than in the BDL + agomir NC group (Figures 8G–I). These findings confirm that the let-7a-5p-TGFBR1/Smad2/Col1a2 regulatory axis potently inhibits α-SMA expression and collagen deposition *in vivo*.

## Discussion

In the past 2 decades, research on epigenetic modification has advanced rapidly (Tzika et al., 2020). Epigenetic alterations include DNA methylation, histone modifications, and changes in noncoding RNA (ncRNA) expression (Thomson and Dinger, 2016). circRNAs, a recently characterized class of ncRNAs, can be classified into three major types: exonic circRNAs (ecircRNAs), circular intronic RNAs (ciRNAs), and exon-intron circRNAs (EIciRNAs) (Zhang et al., 2013). circRNAs perform diverse biological functions, including acting as miRNA sponges, serving as protein sponges or scaffolds, and regulating transcription and translation (Ashwal-Fluss et al., 2014; Du et al., 2016; Yang et al., 2018; Zhang et al., 2018). Among these, exon-derived ecircRNAs frequently function as miRNA sponges because of their enrichment in miRNA response elements (MREs) (Sebastian et al., 2013; Thomas B et al., 2013). By absorbing miRNAs, ecircRNAs prevent them from binding to their target mRNAs, thereby indirectly increasing target-gene expression. In addition, some circRNAs contain multiple MREs and can interact with several miRNAs. Li et al. found that circITCH simultaneously sequesters miR-7, miR-17, and miR-214 (Fang et al., 2015), whereas circHIPK3 regulates cell growth in human cancers by sponging nine different miRNAs (Qiupeng et al., 2016). These observations highlight the complexity of circRNA-mediated regulation. Recent studies have indicated that circRNAs may serve as sensitive biomarkers or potential therapeutic targets in liver diseases (Chien et al., 2020; Guo et al., 2018; Han et al., 2017). Moreover, the roles of circRNAs in HF have gained increasing attention in recent years (Li et al., 2020; Wang et al., 2019; Zhu et al., 2018). Nevertheless, the specific regulatory mechanisms through which circRNAs contribute to HF require further elucidation.

Our study found that circRNAs with excessive lengths or low expression levels could not be specifically amplified. Accordingly, eight highly expressed exon-derived circRNAs of suitable lengths (500–1,500 bp) were selected for preliminary investigation. Among them, hsa_circ_0001377 (circTNK2) was successfully amplified and exhibited the most pronounced increase in TGF-β1-activated LX-2 cells. Sanger sequencing and RNase R digestion assays confirmed the circular structure of hsa_circ_0001377. FISH further confirmed that hsa_circ_0001377 was predominantly localized in the cytoplasm of aHSCs within fibrotic regions. Gain- and loss-of-function experiments demonstrated that hsa_circ_0001377 plays an important regulatory role in HF. Overexpression of hsa_circ_0001377 promoted HSC activation, proliferation, migration, and G1/S-phase transition, whereas its knockdown inhibited HSC activation, proliferation, and migration. These findings suggest that hsa_circ_0001377 may represent a promising therapeutic target for HF.

Analysis using the UCSC Genome Browser revealed that hsa_circ_0001377 is generated through the back-splicing of eight exons of the TNK2 gene. Given that exon-derived ecircRNAs frequently function as miRNA sponges, we further investigated the competing endogenous RNA (ceRNA) mechanism of hsa_circ_0001377. Bioinformatic prediction, qRT-PCR, dual-luciferase reporter, and AGO2-RIP assays collectively demonstrated a direct interaction between hsa_circ_0001377 and let-7a-5p.

The let-7 miRNA family plays critical roles in organ fibrosis. For instance, let-7b contributes to TGF-β1-mediated renal fibrosis by targeting TGFBR1 (Wang et al., 2014), while let-7c-5p is downregulated in renal fibrosis and may be associated with its prognosis and clinical stage (Wang et al., 2020). In this study, we verified that hsa_circ_0001377 directly binds let-7a-5p and further confirmed through dual-luciferase reporter and miRNA pulldown assays that TGFBR1, Smad2, and Col1a2 are direct targets of let-7a-5p.

TGF-β1 and its receptors, TGF-β receptor I (TGFBR1) and TGF-β receptor II (TGFBR2), are important regulators of HF (Lin et al., 2018). The Smad family functions as downstream effectors of TGF-β1 signaling. Upon ligand binding, TGFBR1 and TGFBR2 form a heteromeric receptor complex, in which TGFBR2 phosphorylates TGFBR1. Activated TGFBR1 subsequently phosphorylates Smad2 and Smad3. The phosphorylated Smad2/3 complex translocates to the nucleus, where it regulates the transcription of collagen-related genes (Carl-Henrik and Aristidis, 2016). Accumulating evidence indicates that the TGF-β/Smad signaling pathway plays a central role in HF. Type I collagen, the principal fibrous collagen component of the extracellular matrix produced by HSCs, has a triple-helical structure composed of two α1 chains (Col1a1) and one α2 chain (Col1a2) (Zhansheng et al., 2011). In this study, TGFBR1, Smad2, and Col1a2 were highly expressed in stable hsa_circ_0001377-overexpressing LX-2 cells, and their increased expression was partially reversed by let-7a-5p transfection. Concurrently, the enhanced activation, proliferation, migration, and G1/S-phase transition of stable hsa_circ_0001377-overexpressing LX-2 cells were partially attenuated by let-7a-5p transfection. These findings indicate that hsa_circ_0001377 promotes HF by sequestering let-7a-5p and thereby regulating the expression of TGFBR1, Smad2, and Col1a2.

The preliminary RNA-sequencing analysis was conducted using a limited number of samples. Therefore, an additional cohort of 20 patients with HF was enrolled. hsa_circ_0001377 was highly expressed in HF tissues, and its expression progressively increased with advancing fibrosis stage. Consistently, mmu_circ_0001377 was significantly upregulated in a BDL-induced mouse model of HF. Notably, in both human and mouse fibrotic liver tissues, elevated circ_0001377 expression was accompanied by reduced let-7a-5p expression and increased TGFBR1, Smad2, and Col1a2 expression, indicating that this regulatory axis is conserved across species. Moreover, the BDL model effectively induces HF in mice and closely recapitulates the characteristics of progressive liver fibrosis (Leonel et al., 2002). Because of the technical challenges associated with mmu_circ_0001377 knockdown, mmu-let-7a-5p was administered *via* tail-vein injection. This treatment significantly attenuated liver fibrosis and reduced TGFBR1, Smad2, and Col1a2 expression in BDL mice. Collectively, these findings demonstrate that targeting the circ_0001377/let-7a-5p/TGFBR1, Smad2, and Col1a2 regulatory axis may significantly attenuate HF.

Notably, circRNA expression is typically characterized by temporal and tissue-specific patterns. Using FISH combined with α-SMA co-staining, we observed that hsa_circ_0001377 was significantly enriched in activated hepatic stellate cells (aHSCs) but was undetectable in hepatocytes and bile duct epithelial cells (Figure 1G). Friedman, a distinguished hepatologist, identified the targeting of aHSCs as one of the most promising therapeutic strategies for reversing HF (Friedman, 2015). Studies conducted by Tatiana et al. (2012) and Valery et al. (2008) demonstrated that the aHSC population could potentially be reduced by inducing apoptosis or cellular senescence or by promoting reversion to a quiescent phenotype. Nevertheless, the large number and high volumetric density of parenchymal cells in the liver pose challenges to the precise targeting of HSCs. The abundant localization of hsa_circ_0001377 in the cytoplasm of aHSCs, therefore, provides a natural advantage for its use as a therapeutic target in HF.

Furthermore, HF is a complex process regulated by multiple signaling pathways. Precise regulation can occur at the post-transcriptional level, including through RNA N6-methyladenosine (m6A) modification and ncRNA-mediated mechanisms. According to the competing endogenous RNA (ceRNA) mechanism, circRNAs can sequester multiple miRNAs, while a single miRNA can simultaneously target multiple mRNAs. This intricate circRNA–miRNA–mRNA regulatory network highlights the substantial regulatory potential of circRNAs. In this study, hsa_circ_0001377 was demonstrated to regulate key signaling molecules involved in HF, such as TGFBR1, Smad2, and Col1a2, by sequestering let-7a-5p (graphical abstract). Interestingly, our preliminary experiments showed that other members of the let-7 family, including let-7c-5p, let-7f-5p, and let-7g-5p, also exerted inhibitory effects on HF. Bioinformatic analysis further predicted strong binding between hsa_circ_0001377 and these miRNAs (Supplementary Table S5). Therefore, the broader regulatory functions of hsa_circ_0001377 warrant further investigation.

Taken together, these findings highlight the substantial regulatory role and therapeutic potential of the hsa_circ_0001377/let-7a-5p/TGFBR1, Smad2, and Col1a2 axis in HF.

This study has several limitations. Because directly targeting mmu_circ_0001377 remains technically challenging, mmu-let-7a-5p was used in the *in vivo* experiments. In addition, the effects of hsa_circ_0001377 on Smad2/3 phosphorylation and the nuclear translocation of phosphorylated Smad2/3/4 complexes remain unclear and require further mechanistic validation. These issues will be addressed in future studies.

## Data Availability

The circRNA datasets in this study are available at: https://www.ncbi.nlm.nih.gov/geo/query/acc.cgi?acc=GSE191247. miRNA datasets are available at: https://www.ncbi.nlm.nih.gov/geo/query/acc.cgi?acc=GSE190366.
