# Supplementary material for: hsa_circ_0001377 promotes hepatic fibrosis by sponging let-7a-5p and targeting TGFBR1, Smad2, and Col1a2
Source: Front Pharmacol. 2026 Oct 2;17:1882205. doi: 10.3389/fphar.2026.1882205 (PMC13671924; doi:10.3389/fphar.2026.1882205)
Supplement: Supplementary file 1 [file DataSheet1.doc]

| Age (Y)  Gender (Male)  Hepatocellular carcinoma with fibrosis  Chronic viral hepatitis B (HBV)  Chronic viral hepatitis C (HCV)  Other chronic hepatitis  Biliary cancer with fibrosis  Late-stage liver cirrhosis (liver transplantation)  Other tumors accompanied by fibrosis  Hepatic fibrosis staging  F1  F2  F3  F4 | 56.90±13.01  13（65.00%）  14（70.00%）  10（50%）  2（10%）  2（10%）  2（10%）  1（5%）  3（15%）  2（10.00%）  4（20.00%）  5（25.00%）  9（45.00%） |
| --- | --- |

**Supplementary tables and photos**

**TABLE 1 Clinical parameters of patients with hepatic fibrosis**

**TABLE 2 circRNA primer information**

| Species | CircRNA | | F0R | Primer（5'-3'） |
| --- | --- | --- | --- | --- |
| Homo  mmu | | circALDH9A1  circCUX2  circFGF2  circD2HGDH  circMLXIPL  circTNRC18  circTNK2  （hsa-circ_0001377）  circPER3  mmu-circ_0001377  (mmu_Tnk2_0005000) | 2  0  0  0  0  2  2  0  0 | F1:TGACCGAGAGCGTCCTCTAC  R1: TCTCTGGACAGGGGTTCTGC  F1:GCTCAACCTCAAGACCAACACC  R1: CAGCGAGAAAGTCCGAGTGC  F1: TCTCACAACCAATGAGAGCG  R1: CGTGCTTGATCCACTGGATG  F1:ACATCGTGACTGACCTGCG  R1:GTGGCACGAAGAGGCGA  F1:ACACACCAGCGTTTTGACCA  R1:CGTGGACACCATCCCGTTG  F1:GAGAAGAAAGACTTGGAGTTGGAG  R1:GGGGAGCGGAAAGACAGGT  F1: CACCGTCATCGAGGGAAGAG  R1: AGCCGCAGGAAGTACTGTTG  F1: CCATCTTTTGGGAGGAGCTC  R1: GCTGATATGGCACACTCTTC  F1: CTGTCATCGAGGGAAGAGGCT  R1:AGGCGGGTAATGTTGAGGTC |

Note: The data in the F0R column: 0 denotes back-to-back primers; "F or R + number" indicates that the primer spans the splicing site of the circRNA.

**TABLE 3 mRNA qRT-PCR primer information**

| GENE | Primer（5'-3'） |
| --- | --- |
| TGFBR1  TGFBR3  TAB2  Smad2  FGF5  FGF11  Col1a1  Col1a2  Col3a1  Col4a1  Col4a2  TNK2  gapdh | F: TCAGGTTCTGGCTCAGGTTTA  R: GCCTCACGGAACCACGAA  F: CAGCAAACTTCTCCTTGACAG  R: TTGCTATCTTGAGTTCGGTGA  F: AGGAGGAAGGAATAGAATGATAGC  R: CTTCCCTATCCAATGGCACTA  F: TAGCGTGGCTTGCTGCCTTT  R: GCCCATTCTGCTCTCCTCCG  F: GCAGACAGAGCAGCAGTAGC  R: GGAAACTGCTCTGCTCCAAG  F: CTCTACAGTTCGCCGCATTT  R: GAACGACGCTGGCGGTAG  F: ACGAAGACATCCCACCAATCA  R: CAGATCACGTCATCGCACAAC  F: TGCCTAGCAACATGCCAATC  R: CAGCAAAGTTCCCACCGAGA  F: GAAGGGCAGGGAACAACT  R: CCAGACATCTCTATCCGCAT  F: GAAGGGCAGGGAACAACT  R: CCAGACATCTCTATCCGCAT  F：GAGGCTAAGTGGGACTGACC  R：CACATCAAACTTCTTCACACC  F：CCTCTTCCACCCAATTCCCC  R：AACAGGCTGGTGGGAAAACA  F：GCGGGGCTCTCCAGAACATC  R：TCCACCACTGACACGTTGGC |

**TABLE 4 Information on ecircRNA derived from 8 exons**

| Accession | Chr | Isoform  name | Hosting gene type | Exon | Gene  symbol | Seq  trend |
| --- | --- | --- | --- | --- | --- | --- |
| circRNA12691  circRNA9120  circRNA16992  circRNA12576  circRNA7881  circRNA15398  circRNA6354  circRNA6522 | chr1  chr12  chr10  chr2  chr7  chr3  chr3  chr1 | ENST00000354775  ENST00000261726  ENST00000357555ENST00000436747  ENST00000345114  ENST00000399537  ENST00000381916  ENST00000361923 | mRNA  mRNA  mRNA  mRNA  mRNA  mRNA  mRNA  mRNA | 9  7  11  5  9  8  8  5 | ALDH9A1  CUX2  FGF2  D2HGDH  MLXIPL  TNRC18  TNK2  PER3 | up  up  up  up  up  up  up  up |

Note: In this table, circRNA is designated as the RNA-seq sequencing code（circRNA6354

=circTNK2=hsa-circ_0001377）.

**TABLE 5 Prediction of hsa-circ_0001377-targeted miRNAs**

| miRNA | miRanda Score | miRanda Energy (kCal/Mol) |
| --- | --- | --- |
| hsa-let-7a-5p | 161 | -24.17 |
| hsa-let-7c-5p | 156 | -21.35 |
| hsa-let-7f-5p | 161 | -22.25 |
| hsa-let-7g-5p | 165 | -28.200001 |
| hsa-miR-187-3 | 145 | -17.799999 |
| hsa-miR-214-3p | 148 | -19.59 |
| hsa-miR-125b-5p | 143 | -23.74 |
| hsa-miR-128-1-5p | 141 | -28.809999 |
| hsa-miR-145-5p | 141 | -23.530001 |
| hsa-miR-20a-5p | 157 | -25.59 |
| hsa-miR-23a-5p | 146 | -27.290001 |
| hsa-miR-24-3p | 153 | -20.23 |
| hsa-miR-125a-5p | 144 | -22.360001 |
| hsa-miR-134-3p | 145 | -19.969999 |
| hsa-miR-149-5p | 147 | -13.88 |
| hsa-miR-185-3p | 140 | -26.25 |
| hsa-miR-188-3p | 145 | -21.52 |
| hsa-miR-296-5p | 145 | -19.51 |
| hsa-miR-370-3p | 150 | -25.85 |
| hsa-miR-382-5p | 147 | -26.33 |
| hsa-miR-328-3p | 140 | -17.01 |
| hsa-miR-342-5p | 146 | -23.65 |
| hsa-miR-323a-5p | 152 | -22.370001 |
| hsa-miR-345-3p | 142 | -27.92 |
| hsa-miR-431-3p | 147 | -22.450001 |
| hsa-miR-329-5p | 145 | -27.98 |
| hsa-miR-486-3p | 146 | -22.52 |
| hsa-miR-193b-5p | 145 | -24.469999 |
| hsa-miR-505-5p | 143 | -30.43 |
| hsa-miR-608 | 145 | -27.57 |
| hsa-miR-615-5p | 142 | -28.809999 |
| hsa-miR-617 | 148 | -22.200001 |
| hsa-miR-623 | 149 | -30.700001 |
| hsa-miR-636 | 141 | -18.969999 |

**FIGURE 1 Construction of hsa-circ_0001377 overexpression lentiviral vector**

**(A)** Amplification product of target gene fragment, #1 represents the target amplification product, and #2 is a 5kb Marker; **(B)** Colony PCR identification, #1 is the negative control (ddH2O), #2 is the empty vector self-ligation control, #3 is the positive control (GAPDH), #4 is a 5kb Marker, and #5~#12 represent transformants; **(C)** Sequencing results of positive clone transformants

**FIGURE 2 Melting curves of circRNA**

The melting curves of circCUX2, circFGF2, circD2HGDH, circTNK2, and circPER3 exhibit a significant single-peak distribution

**FIGURE 3 RNase R digestion experiment**

Compared to the linear GAPDH,hsa- circ_0001377 exhibits stronger RNase R resistance

**FIGURE 4 Viral titer detection of circ_0001377 overexpression vector (200×)**

1E1 μL, 1E-0 μL, and 1E-1 μL represent virus addition volumes of 10 μL, 1 μL, and 10-1 μL, respectively

**FIGURE 5 qRT-PCR assay showed the linear mRNA of the TNK2 gene expression in the si-NC and si-hsa-circ_0001377 groups.**

**FIGURE 6 qRT-PCR experiment shows the expression level of α-SMA and Col1a1 in each group**

**FIGURE 7 CCK-8 assay**

The CCK-8 assay showed the OD450 fold values of LX-2 cells in each group from 24h to 120h, * vs let-7a-5p, # vs mimic NC;
